# Potential mechanisms linking probiotics to diabetes: a narrative review of the literature: Mecanismos potenciais ligando probióticos a diabetes: uma revisão narrativa da literatura

**DOI:** 10.1590/1516-3180.2016.0311271216

**Published:** 2017-04-20

**Authors:** Maryam Miraghajani, Somayeh Shahraki Dehsoukhteh, Nahid Rafie, Sahar Golpour Hamedani, Sima Sabihi, Reza Ghiasvand

**Affiliations:** I PhD. Doctoral Student, Cancer Research Center, Shahid Beheshti University of Medical Sciences, Tehran, Iran.; II MSc. Coach, Department of Statistics, Faculty of Sciences, Zabol University, Zabol, Iran.; III MSc. Master’s Student, Food Security Research Center, Department of Community Nutrition, School of Nutrition and Food Science, Isfahan University of Medical Sciences, Isfahan, Iran.; IV PhD. Professor, Food Security Research Center, Department of Community Nutrition, School of Nutrition and Food Science, Isfahan University of Medical Sciences, Isfahan, Iran.

**Keywords:** Molecular mechanisms of pharmacological action, Probiotics, Diabetes mellitus, Review, Microbiota

## Abstract

**CONTEXT AND OBJECTIVE:**

Some studies have suggested a wide range of possible mechanisms through which probiotics may play a role in diabetes prevention and treatment. However, the underlying mechanisms are not fully understood. We conducted this study to review the potential mechanisms suggested for the effect of probiotics in diabetes.

**DESIGN AND SETTING:**

Narrative review conducted at the Food Security Research Center of Isfahan.

**METHODS:**

A search in the electronic databases MEDLINE (PubMed), Cochrane Library, Web of Science and Google scholar was performed up to October 2016.

**RESULTS:**

The initial search yielded 1214 reports. After removing duplicates, 704 titles and abstracts were screened. Finally, out of 83 full-text articles that were reviewed for eligibility, 30 articles were included in the final analysis. The anti-diabetic mechanisms for probiotics reported encompass intraluminal and direct effects on the intestinal mucosa and microbiota (n = 13), anti-inflammatory and immunomodulatory effects (n = 10), antioxidative effects (n = 5), effects on endoplasmic reticulum (ER) stress and expression of genes involved in glucose homeostasis and insulin resistance (n = 6), with some studies pointing to more than one mechanism.

**CONCLUSION:**

The results may throw some light on the capacity of probiotics as a novel approach towards controlling diabetes. However, further human studies are warranted to elucidate and confirm the potential role of probiotics in diabetes prevention and treatment. Also, it needs to be ascertained whether the effectiveness of probiotics in diabetes prevention and treatment is dependent on the strain of the microorganisms.

## RESUMO

**CONTEXTO E OBJETIVO:**

Alguns estudos têm sugerido ampla gama de possíveis mecanismos, pelos quais os probióticos podem desempenhar um papel na prevenção e tratamento do diabetes. No entanto, os mecanismos subjacentes não são totalmente compreendidos. Realizamos este estudo para revisar os possíveis mecanismos sugeridos para o efeito dos probióticos na diabetes.

**TIPO DE ESTUDO E LOCAL:**

Revisão narrativa conduzida no Food Security Research Centro de Isfahan.

**MÉTODOS:**

Busca sistemática nas bases de dados eletrônicas MEDLINE (PubMed), Cochrane Library, Web of Science e Google scholar até outubro de 2016.

**RESULTADOS:**

A busca inicial resultou em 1.214 artigos. Após a remoção de duplicatas, foram pesquisados 704 títulos e resumos. Finalmente, de 83 artigos completos revisados para elegibilidade, 30 foram incluídos na análise final. Os mecanismos antidiabéticos relatados dos probióticos abrangem efeitos intraluminais e diretos na mucosa e microbiota intestinal (n = 13), efeitos anti-inflamatórios e imunomoduladores (n = 10), efeitos antioxidativos (n = 5), efeitos sobre o estresse de retículo endoplasmático (RE) e expressão de genes envolvidos na homeostase da glicose e resistência à insulina (n = 6), com alguns estudos apontando para mais de um mecanismo.

**CONCLUSÃO:**

Os resultados podem lançar alguma luz sobre os probióticos como uma nova abordagem no controle do diabetes, no entanto, mais estudos em humanos são justificados para elucidar e confirmar o papel potencial dos probióticos na prevenção e tratamento do diabetes. Além disso, deverá ser determinado se a eficácia dos probióticos na prevenção e tratamento do diabetes é dependente da cepa dos microrganismos.

## INTRODUCTION

Probiotics are live microorganisms that may exert beneficial effects regarding the sufficiency of consumption via their impact on the microbial balance of the gut.^1^ The most commonly used probiotics are *Lactobacillus*, *Bifidobacterium* and *Saccharomyces boulardii*, which have different effects depending on the dosage, length of therapy and administration route.^2^

Given the influence of the gut microbiota on metabolic conditions including diabetes and on improving host metabolism, the concept of manipulating the gut microbiota has gained considerable interest over recent years. Use of probiotics has been suggested as one of the approaches towards modifying the clonal flora.^3^

Diabetes mellitus is a chronic metabolic disease with major complications largely influenced by glycemic measures.^1^ The Global Burden of Disease 2015 study (GBD 2015) showed that diabetes was among the leading causes of years of life lost (YLLs) in most regions.^2^ Also, diabetes was shown to be a leading cause of disability-adjusted life years (DALYs), for which the observed burden exceeded expected levels in many localities.^3^ The rise in diabetes prevalence is set to pose one of the most important challenges to healthcare systems over the coming years.^4^

A growing body of evidence suggests that favorable associations exist between probiotic consumption and metabolic profile among diabetes subjects.^5^ However, the potential mechanisms underlying the effects of probiotics on glycemia-related parameters are not fully understood. One of the main mechanisms postulated may involve increased glucagon-like peptide 1 (GLP-1) secretion from enteroendocrine L-cells to improve carbohydrate metabolism, decrease glucotoxicity and increase insulin sensitivity of target cells.^6^ Other proposed mechanisms to explain the action of probiotics on diabetes relate to anti-inflammatory, antioxidant and immunomodulatory effects and alteration of the expression of some genes involved in diabetes.^7^^,^^8^^,^^9^^,^^10^

Moreover, probiotic intake affects the structure of the gut flora, which might improve the integrity of the intestinal epithelium, weaken the immune responses and diminish the toll-like receptor 4 pathway, which in turn reduces pro-inflammatory signaling and enhances insulin sensitivity.^11^^,^^12^

Given the various statements regarding the effects of probiotics on diabetes that have been made, the aim of the present study was to focus on possible mechanisms for probiotics that might explain some of their beneficial effects in relation to diabetes, in the form of a review.

## OBJECTIVE

The aim of the present study was to focus on possible mechanisms for probiotics that might explain some of their beneficial effects in relation to diabetes, in the form of a narrative review. 

## METHODS

### Search strategy

A search of the electronic databases MEDLINE (via PubMed) and Cochrane Library (via Wiley) and the electronic repositories Web of Science and Google Scholar was performed. The search was last performed in October 2016, using combinations of search terms including “probiotics” OR “probiotic” OR “lactic acid bacteria” OR “lactobacillus” OR “lactobacilli” OR “bifidobacterium” OR “bifidobacteria” AND “diabetes mellitus”, without any restrictions, in order to find studies focusing on the mechanisms linking probiotics with diabetes.

### Eligibility criteria

Studies were included if they assessed the effect of a single or combination of live probiotics on diabetes. On the other hand, studies presented only as abstracts with no full-text available, non-English literature, studies involving patients with other metabolic diseases such as obesity or hypercholesterolemia, studies with no probiotic genus/strains reported, studies using synbiotics (i.e. probiotics combined with prebiotics), study protocols, pilot studies, letters, editorials, obviously irrelevant studies and studies that included non-diabetic patients or animals were all excluded.

### Selection strategy

The eligibility of all potential studies identified for inclusion was independently assessed by two reviewers. Discrepancies regarding study inclusion were resolved through discussion with a third reviewer. Initially, titles and abstracts were verified and then an assessment of full texts was conducted. The reference lists of eligible articles or relevant review papers were screened for other eligible papers.

### Data extraction

Study characteristics from eligible articles such as the first author’s name, year of publication, study design, subjects or animal models, probiotic strain and suggested mechanisms for probiotics on diabetes were extracted by two authors. The details of all eligible articles are outlined in Table 1.^10^^,^^11^^,^^12^^,^^13^^,^^14^^,^^15^^,^^16^^,^^17^^,^^18^^,^^19^^,^^20^^,^^21^^,^^22^^,^^23^^,^^24^^,^^25^^,^^26^^,^^27^^,^^28^^,^^29^^,^^30^^,^^31^^,^^32^^,^^33^^,^^34^^,^^35^^,^^36^^,^^37^^,^^38^^,^^39^

**Table 1:**
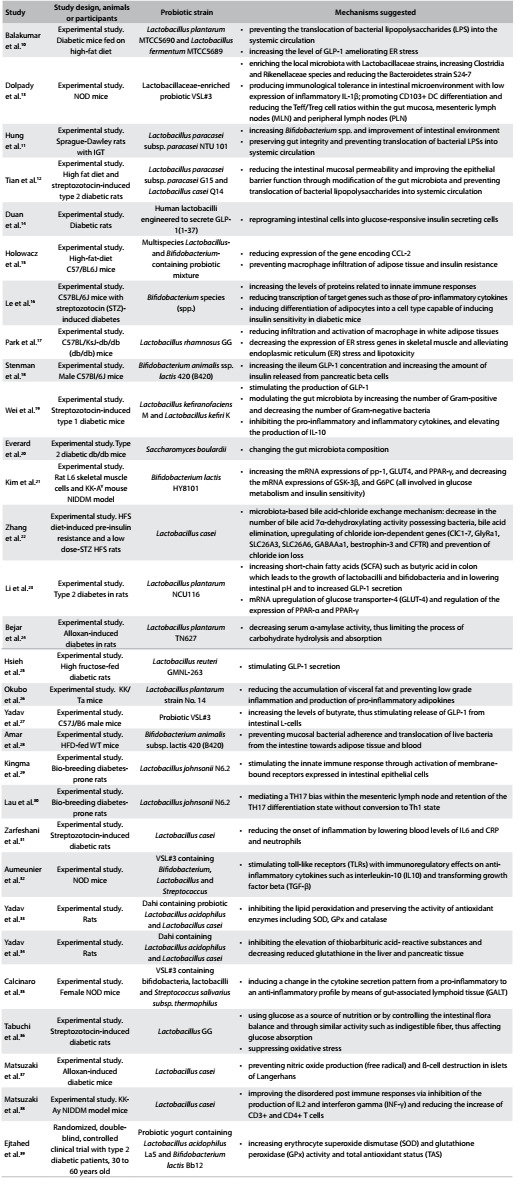
Characteristics of the studies included

## RESULTS

**Figure 1:**
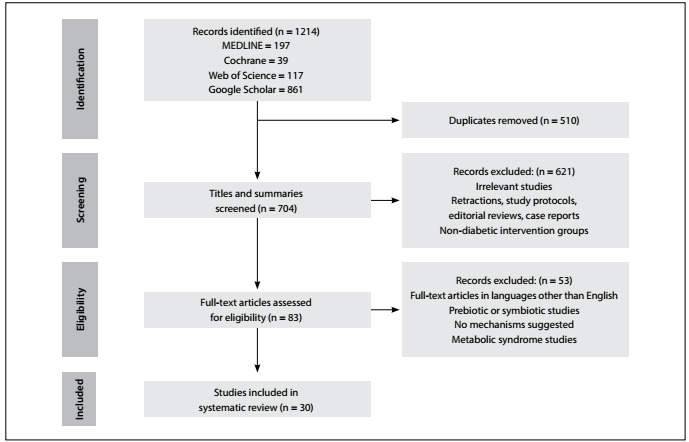
Flow chart of study selection process.

Our initial search retrieved 1,214 articles. After removing duplicates, 704 titles and abstracts were screened. Then, from among these articles, 83 full texts were assessed for eligibility. Finally, 30 studies were included in this review. A flowchart of the study selection process is illustrated in Figure 1.

### Local effects of probiotics in the intestine

Endotoxemia (increased circulatory levels of bacterial lipopolysaccharides) has been identified as a triggering factor for insulin resistance in mice and suppression of endotoxemia by probiotic supplementation is considered to be a protective mechanism.^40^ In this regard, Balakumar et al.^10^ stated that probiotic interventions increased the gene expression profile of the intestinal tight junction markers and gut integrity, thereby preventing translocation of bacterial lipopolysaccharides (LPS) into the systemic circulation.

Furthermore, treatment with *Lactobacillus paracasei subsp. paracasei* NTU 101 may lessen the risk of type 2 diabetes mellitus through increased levels of *Bifidobacterium spp.* and improvement of the intestinal environment. This would preserve gut integrity and prevent translocation of bacterial lipopolysaccharides into the systemic circulation.^18^

Similarly, presence of *Lactobacillus paracasei subsp. paracasei* G15 and *Lactobacillus casei* Q14 in the gut has shown a clear correlation with reduced intestinal mucosal permeability and improved epithelial barrier function, through modification of the gut microbiota. In turn, this has been shown to lower the circulating levels of LPS and inflammatory cytokines, including interleukin (IL)-1β and IL-8, and possibly to alleviate the inflammatory status and islet β-cell dysfunction.^19^

Treatment with the probiotic *Bifidobacterium animalis subsp. lactis* 420 (B420) in another study^14^ led to protection against diabetes through prevention of mucosal bacterial adherence and translocation of live bacteria from the intestine towards adipose tissue and blood, which caused inflammation and insulin resistance.

Incretins, especially glucagon-like peptide 1 (GLP-1) secreted by intestinal L-cells, are a group of metabolic hormones that inhibit postprandial hyperglycemia by increasing the amount of insulin released from pancreatic beta cells.^40^ Several studies^10^^,^^18^^,^^25^ have shown that the beneficial effects of probiotic interventions on glucose tolerance and insulin sensitivity were related to increased levels of GLP-1.

Also, administration of *Lactobacillus kefiranofaciens M* and *Lactobacillus kefiri K* was found to stimulate GLP-1 production, with a concomitant decrease in the numbers of Gram-negative bacteria, which could trigger inflammation.^19^

The influence of human lactobacilli engineered to secrete GLP-1 on hyperglycemia has been investigated by Duanet al.^14^ They showed that these lactobacilli reprogram intestinal cells into glucose-responsive insulin-secreting cells and that they therefore had the ability to ameliorate hyperglycemia and diabetes.

Moreover, in some studies, the effect of probiotics on diabetes has been linked to increases in the levels of short-chain fatty acids (SCFAs), especially butyrate in the colon.^23^^,^^27^ SCFAs are probably key components in the growth of lactobacilli and bifidobacteria and in lowering intestinal pH. All of these are expected to have beneficial effects on diabetes. In addition, SCFAs have been linked to increased GLP-1 secretion in both animal and human models.

Local changes to the intestinal environment and microbiota have been mentioned as another mechanism relating probiotics to diabetes prevention and treatment. *Saccharomyces boulardii* significantly changes the gut microbiota composition with an increased proportion of Bacteroidetes and decreased quantities of organisms in the phyla Firmicutes, Proteobacteria and Tenericutes. These phyla have been previously correlated with type 2 diabetes in mice.^20^

Dolpady et al.^13^ also demonstrated prevention of type 1 diabetes (T1D) through enriching the local microbiota with Lactobacillaceae strains and through inducing substantial modifications in the microbiota composition, with increased levels of species of Clostridia and Rikenellaceae and decreased levels of the Bacteroidetes strain S24-7, when a Lactobacillaceae-enriched VSL#3 probiotic was administered. In addition, these modifications generated a protolerogenic intestinal microenvironment with low expression of inflammatory IL-1β. The VSL#3-induced protolerogenic microenvironment promotes CD103+ dendritic cell differentiation and reduces T effectors/T regulatory cell (Teff/Treg) ratios within the gut mucosa, mesenteric lymph nodes (MLN) and peripheral lymph nodes (PLN), which results in autoimmune diabetes prevention.

Pancreatic inflammation caused by type 1 diabetes results in leakage of a-amylase into the bloodstream, thus eliciting higher levels of serum pancreatic a-amylase, a key enzyme involved in carbohydrate digestion. Administration of L. plantarum TN627 to diabetic rats was found to significantly decrease serum α-amylase activity, thus limiting the process of carbohydrate hydrolysis and absorption. Consequently, beneficial effects were observed on the glycemic index.^24^

### The effects of probiotics on the inflammatory and immune response pathways

Altered production or function of circulating innate immune proteins, cellular pattern-recognition receptors and inflammatory cytokines have been linked to insulin resistance and diabetes.^41^

*Lactobacillus kefiranofaciens* M and *lactobacillus kefiri* K were reported to mitigate progression of type 1 diabetes through inhibiting pro-inflammatory and inflammatory cytokines and elevating the production of IL-10. IL-10 inhibits the levels of pro-inflammatory cytokines (tumor necrosis factor-alpha) and Th1 cytokines (IL-1β, IL-2, IL-6) and prevents β cell destruction.^19^

Moreover, administration of *Bifidobacterium spp.* increased the levels of innate immune response proteins, including IκB kinase alpha (IKKα), nuclear factor-kappa B inhibitor alpha (IκBα), extracellular-signal-regulated kinase 2 (ERK2) and protein kinase B (Akt). Akt may affect IKKα and even result in activation of IκBα, which may in turn inhibit the effects of NF-κBand, thus leading to reduced transcription of target genes such as those of pro-inflammatory cytokines. On the other hand, ERK, a widely-expressed protein kinase, is an intracellular signaling molecule involved in functions relating to regulation of cell proliferation, differentiation and survival. Increased ERK2 levels may induce differentiation of adipocytes into a cell type capable of inducing insulin sensitivity in diabetic mice fed with *Bifidobacterium spp*.^16^

Furthermore, *Lactobacillus rhamnosus* GG (LGG) treatment was shown^17^ to reduce infiltration and activation of macrophages, which is critical for initiation and amplification of chronic inflammation in white adipose tissues. Hence, the insulin-sensitizing effect of LGG may occur through alleviating this inflammatory pathway.

Another study^26^ indicated that administration of *Lactobacillus plantarum* No. 14 prevents development of insulin resistance, mainly through reducing accumulations of visceral fat, which prevents production of pro-inflammatory adipokines. Pro-inflammatory adipokines interfere with the insulin-signaling pathway of peripheral tissues and facilitate development of insulin resistance.

In addition, there is evidence that oral treatment with VSL#3, a probiotic compound containing bifidobacteria, lactobacilli and *Streptococcus salivarius subsp*. *thermophilus*, induces a change in the cytokine secretion pattern from a pro-inflammatory to an anti-inflammatory profile in the gut-associated lymphoid tissue (GALT), which is associated with qualitative modification of islet-specific destructive autoimmunity and, possibly, diabetes prevention.^35^

Consistent with the abovementioned data, protective action by *Lactobacillus casei* in relation to diabetes was correlated with less frequent onset of inflammation, through lowered levels of IL6, CRP and neutrophils in blood.^31^*Lactobacillus casei* also has the potential to decrease blood glucose levels through improvement of disordered post-immune responses via inhibition of production of IL2 and interferon gamma (INF-γ) and reduction of the increases in CD3+ and CD4+ T cell counts.^38^

Kingma et al.^29^ showed that *Lactobacillus johnsonii* (Ljo) N6.2 stimulates the innate immune response through activation of the membrane-bound receptors expressed in intestinal epithelial cells. These receptors activate type 1 interferon (INF), which are key players in innate immunity. Therefore, a higher state of immunological activation would be achieved, thereby preventing diabetes. Moreover, this strain inhibits type 1 diabetes through mediating T-helper 17 (Th17) bias within the mesenteric lymph nodes. Retention of the Th17 differentiation state, without conversion to a Th1 state, which is critical to diabetogenesis, prevents or delays the onset of type 1 diabetes.^30^

Stimulation of toll-like receptors (TLRs), which have immuno-regulatory effects on anti-inflammatory cytokines, can prevent the onset of autoimmune diseases. TLR-mediated effects of probiotics involve immune-regulatory cytokines such as interleukin IL-10 and transforming growth factor (TGF)-β and some regulatory T cells, under the experimental conditions that result in protection from spontaneous diabetes.^32^

### The effects of probiotics on oxidative stress 

In diabetes, the free radicals that are generated cause lipid peroxidation and malondialdehyde (MDA) production. Moreover, the activity levels of reactive oxygen species scavengers are lower in patients with diabetes. Therefore, improvement of oxidative stress status may contribute towards diabetes management.^42^^,^^43^ Tabuchi et al.^36^ showed that *Lactobacillus* GG lowered the level of MDA per gram of liver weight, which conferred suppression of oxidative stress and improved glucose tolerance.

Other authors concluded that the inhibitory effect of *Lactobacillus casei* on the incidence of diabetes was partially dependent on prevention of nitric oxide production, given that this is a free radical that is involved in the ß-cell destruction process in islets of Langerhans.^37^^,^^44^

On the other hand, foods containing probiotics have been shown to protect against indices relating to diabetes. In one study, probiotic yogurt consumption increased the activity levels of erythrocyte superoxide dismutase (SOD) and glutathione peroxidase (GPx), which scavenge free radicals, and improved the total antioxidant status (TAS).^39^

Another mechanism that was proposed to explain the action of fermented milk products containing probiotic bacteria on diabetes was through diminishing the elevation of thiobarbituric acid-reactive substances and increasing glutathione levels in the liver and pancreatic tissues of diabetic rats. These findings indicated that this drink had good antioxidant properties.^33^

Probiotic milk has consistently been found to exert antioxidant effects through inhibiting lipid peroxidation and preserving the activity of antioxidant enzymes, including SOD, GPx and catalase (CAT).^34^

### The effects of probiotics on gene expression

Some studies on interactions between probiotics and gene expression have suggested that type 2 diabetes in rats is ameliorated through mRNA upregulation of glucose transporter-4 (GLUT-4) through *Lactobacillus plantarum* NCU116 treatment.^23^ This has a critical role in glucose uptake.^45^ Moreover, NCU can regulate glucose homeostasis and insulin sensitivity in diabetic rats via regulating PPAR-α and PPAR-γ gene expression. These genes play key roles in inflammation and glucose homeostasis.^46^

*Bifidobacterium* spp. also has an impact on enhanced expression of proteins involved in the insulin-signaling pathway, including IR-β, IRS-1 and Akt. This results in improved glucose uptake and blood glucose reduction.^16^

Zhang et al.^22^ postulated that prevention of the onset of type 2 diabetes through using *L. casei* Zhang may occur via a microbiota-based bile acid-chloride exchange mechanism. Hyperglycemia relates to high levels of plasma bile acids and urine chloride ion loss. High intracellular chloride ion levels in β-cells of the pancreas are essential for the electrical activity of the β-cell membrane and for insulin release. *L. casei* Zhang administration was found to cause a decrease in the quantity of bacteria with bile acid 7α-dehydroxylating activity and, therefore, bile acid elimination was enhanced. In turn, chloride ion loss was significantly prevented by *L. casei* via upregulation of chloride ion-dependent genes (ClC1-7, GlyRa1, SLC26A3, SLC26A6, GABAAa1, bestrophin-3 and CFTR).

In addition, discovery of the antidiabetic activity of *Bifidobacterium lactis* HY 8101 has shed new light on the mechanisms for probiotics and their importance in diabetes.^21^ Its antidiabetic activity occurs through increasing the mRNA expression of pp-1 (glycogen synthesis-related enzymes), GLUT4 (glucose uptake-related genes) and PPAR-γ (insulin sensitivity-related genes) and decreasing the mRNA expression of GSK-3β (glycogen synthesis-related enzymes) and G6PC (gluconeogenesis-related enzymes), which are all involved in glucose metabolism and insulin sensitivity.

Another investigation^15^ also provided evidence that a multispecies mixture of probiotics containing *Lactobacillus* and *Bifidobacterium* reduced expression of the gene encoding CCL-2. The latter is an important chemokine for macrophage infiltration of adipose tissue and contributes towards insulin resistance.^47^

Finally, endoplasmic reticulum (ER) stress has been mentioned as one of the main causes of development of inflammation and insulin resistance. ER stress appears to act directly as a negative modulator of the insulin signaling pathway, but also indirectly by promoting lipid accumulation.^48^ Two studies^10^^,^^17^ showed that probiotic interventions alleviated lipotoxicity and ER stress gene expression in skeletal muscle, which resulted in improvement of glucose tolerance.

## DISCUSSION 

One significant question regarding clinical use of probiotics is the mechanism underlying the wide range of actions. However, the increasing number of studies that are being conducted with the aim of establishing probiotic mechanisms relating to diabetes conditions indicate that there is a promising future for probiotics in treating this disease. To the best of our knowledge, this is the first review on the mechanisms of probiotic function relating to diabetes. It is hoped that gaining a mechanistic understanding of probiotic action will provide the rationale to support development of new hypothesis-driven studies to define the clinical efficacy of preventive, adjunctive or alternative treatments for diabetes. Also, such efforts could suitably help in selecting strains for specific investigation and applications under these conditions and may uncover novel probiotic functions.

The mechanisms suggested have mostly involved intraluminal and direct effects on intestinal mucosa and microbiota (13 studies). Suppression of endotoxemia, stimulation of secretion of short chain fatty acids (SCFAs) and incretines, and local changes to the gut environment and microbiota were major effects detailed in the present review. In addition, anti-inflammatory and immunomodulatory effects were reported in 10 studies. Prevention of free radical production, increased activity of antioxidant enzymes and inhibition of peroxidation were reported as the main antioxidant effects of probiotics in relation to diabetes (five studies). Finally, six studies suggested that probiotics might have effects through altering the expression of genes involved in ER stress and glucose homeostasis and insulin resistance.

The strengths of this review include its use of an outcome classification for different possible mechanisms of probiotics in relation to diabetes. However, several limitations need to be taken into account in interpreting our findings. It should be mentioned that, except for one study, all of these mechanisms have been verified in animal studies. Moreover, it seems that such effects depend on the type of bacteria, dose and duration of consumption, manner and frequency of administration, environmental factors and complex interactions between probiotics, cells and metabolic pathways that are rarely mediated by a single mechanism.^49^

In addition, it is important to take into consideration the risk of bias across different studies, such as publication, performance and reporting bias, along with potential conflicts of interest. Such factors might limit the ability to draw robust conclusions from these studies. Given that we only had limited access to some databases such as Embase, and that studies not reported in English were excluded, it is possible that more rigorous reporting of study results would improve the quality of the evidence in further studies.

Nonetheless, elucidation of the mechanisms linking the microbiome to diabetes can provide a rational basis for dietary consumption of probiotic microorganisms in relation to diabetes. In addition, evaluation of the mechanism of action for probiotics both in healthy subjects and in diabetic patients, so as to address the influence of these microorganisms on gene expression for different pathways, is needed in order to better understand the role that probiotics might have in prevention and treatment of diabetes.

## CONCLUSIONS

In conclusion, there is some evidence suggesting various potential mechanisms of action for probiotics in relation to diabetes prevention and treatment. Further studies are needed to confirm the underlying pathways involved in the beneficial effects from each strain, along with assessment of other confounding factors.
